# Novel Peptides Based on HIV-1 gp120 Sequence with Homology to Chemokines Inhibit HIV Infection in Cell Culture

**DOI:** 10.1371/journal.pone.0014474

**Published:** 2011-01-11

**Authors:** Oleg Chertov, Ning Zhang, Xin Chen, Joost J. Oppenheim, Jacek Lubkowski, Connor McGrath, Raymond C. Sowder, Bruce J. Crise, Anatoli Malyguine, Michele A. Kutzler, Amber D. Steele, Earl E. Henderson, Thomas J. Rogers

**Affiliations:** 1 Protein Chemistry Laboratory, Advanced Technology Program, SAIC-Frederick, Inc., National Cancer Institute at Frederick, Frederick, Maryland, United States of America; 2 Laboratory of Molecular Immunoregulation, National Cancer Institute at Frederick, Frederick, Maryland, United States of America; 3 Macromolecular Crystallography Laboratory, National Cancer Institute at Frederick, Frederick, Maryland, United States of America; 4 Target Structure-Based Drug Discovery Group, Frederick, Maryland, United States of America; 5 AIDS and Cancer Virus Program, SAIC-Frederick, Inc., Frederick, Maryland, United States of America; 6 Clinical Services Program, SAIC-Frederick, Inc., Frederick, Maryland, United States of America; 7 Department of Pharmacology, Fels Institute for Cancer Research and Molecular Biology, Center for Substance Abuse Research, Temple University School of Medicine, Philadelphia, Pennsylvania, United States of America; Tsinghua University, China

## Abstract

The sequential interaction of the envelope glycoprotein of the human immunodeficiency virus type 1 (HIV-1) with CD4 and certain chemokine coreceptors initiates host cell entry of the virus. The appropriate chemokines have been shown to inhibit viral replication by blocking interaction of the gp120 envelope protein with the coreceptors. We considered the possibility that this interaction involves a motif of the gp120 that may be structurally homologous to the chemokines. In the amino acid sequences of most chemokines there is a Trp residue located at the beginning of the C-terminal α-helix, which is separated by six residues from the fourth Cys residue. The gp120 of all HIV-1 isolates have a similar motif, which includes the C-terminal part of a variable loop 3 (V3) and N-terminal part of a conserved region 3 (C3). Two synthetic peptides, derived from the relevant gp120 sequence inhibited HIV-1 replication in macrophages and T lymphocytes in sequence-dependent manner. The peptides also prevented binding of anti-CXCR4 antibodies to CXCR4, and inhibited the intracellular Ca^2+^ influx in response to CXCL12/SDF-1α. Thus these peptides can be used to dissect gp120 interactions with chemokine receptors and could serve as leads for the design of new inhibitors of HIV-1.

## Introduction

The envelope glycoprotein of the human immunodeficiency virus type 1 (HIV-1) mediates the fusion of viral and host cell membranes necessary for virion entry [1]. The envelope glycoprotein of HIV-1 is produced by the enzymatic cleavage from the gp160 precursor protein with formation of the external gp120 and the transmembrane gp41 proteins [2]. Several studies have identified the amino acid residues of gp120 that are responsible for the specific interaction with CD4, the primary receptor for HIV-1 [3]–[6]. It is likely that a conformational change occurs in gp120 following the binding to CD4, and this exposes a binding site(s) for the chemokine receptor/viral coreceptor [5]. The major viral coreceptors are CXCR4, the receptor for stromal derived factor-1 (CXCL12/SDF-1α), and CCR5, a receptor for several chemokines including macrophage inflammatory protein-1β (CCL4/MIP-1β) [7]. Recent studies suggest that the association of the CD4-gp120 complex with the viral coreceptor leads to a rearrangement of gp120 that allows the interaction of the gp41 envelope protein subunit with the host cell membrane and viral entry [8], [9]. Since chemokine receptors have presumably evolved for the selective binding of chemokines, we proposed that a region of the viral glycoprotein gp120, responsible for recognition of coreceptors, might have a structural element similar to that found in the respective chemokine ligand. In the amino acid sequences of most chemokines, there is a Trp residue located at the beginning of C-terminal α-helix that is separated by six residues from the fourth Cys residue. The gp120 of all HIV-1 isolates have a very similar motif adjacent to the V3 loop. We hypothesized that this region of gp120 may directly interact with chemokine receptors. The synthesized peptides based on the relevant gp120 sequence were found to interfere with chemokine receptor function and inhibit HIV replication in susceptible cells.

## Materials and Methods

### Synthetic peptides

Peptides 15D, 15K and 15KS were synthesized by Macromolecular Resources (Fort Collins, CO). The peptides were purified by reverse-phase HPLC, and their homogeneity was confirmed by mass-spectrometry.

### Computer modeling

A model of the gp120 fragment (residues 331–340) was derived by superimposing heavy atoms onto the corresponding heavy atoms of CCL4 residues 51–60, which is in a helical conformation (the atomic coordinates were obtained from X-ray structure of CCL4, pdb code 1 hum). Starting with a helical conformation of the gp120 fragment, backbone atoms and homologous side chain atoms were template forced onto the CCL4 structure during this protocol. The resulting gp120 model helix was optimized using a constrained protocol of sampled molecular dynamics structures followed by conjugate gradients minimization and selection of minima under a consistent valence force field (CVFF) [10]–[12].

### Cells and culture conditions

CEMx174 cell line was obtained from ATCC (Rockville, MD). HEK293/CXCR4 and HEK293/CCR5 cell lines were kindly provided by O.M.Zack Howard [13]. Cells were cultured in RPMI-1640 medium (BioWhittaker, Walkersville, MD) containing 10% fetal bovine serum (FBS; HyClone, Logan, UT), 2 mM glutamine, 100 units/ml penicilin and streptomycin (Quality Biologicals, Gaithersburg, MD) at 37°C in a humidified 5% CO_2_ atmosphere.

### Preparation of monocytes, monocyte-derived macrophages and T cells

Peripheral blood mononuclear cells (PBMCs) were obtained from the whole blood of seronegative donors and isolated by Ficoll-Paque Plus (Pharmacia Biotech, Piscataway, NJ) using standard density gradient centrifugation techniques. Enriched populations of monocytes or CD4-positive T cells were prepared by magnetic bead separation using anti-CD14 or anti-CD4 microbeads (Miltenyi Biotec, Inc, Auburn, CA) in RPMI-1640 medium supplemented with 10% heat-inactivated, low-endotoxin fetal calf serum (Hyclone Laboratories, Logan, UT), 10 µg/mL gentamicin, and 1 mM L-glutamine.

Monocyte-derived macrophages (MDM) were generated from adherent human peripheral blood mononuclear cells by culture for 7 days with M-CSF (100 ng/ml). Cultures were maintained in RPMI-1640 medium (Life Technologies, Rockville, MD) supplemented with 10% heat-inactivated endotoxin-free FCS (Hyclone, Logan, UT), 10 µg/ml gentamicin, and 1 mM glutamine.

There was no toxicity detected for any of the peptides using trypan blue dye exclusion. Cells treated at each dose of peptide were assessed for evidence of toxicity at 4 hours and 3 days following peptide administration, at each dose employed. We observed no detectable cytotoxicity of the peptides on the cells even at 50 µM concentration.

### HIV-1

The R5 JRFL and X4 IIIB strains of HIV-1 were obtained from the National Institute of Allergy and Infectious Diseases AIDS Research and Reference Reagent Program (Rockville, MD). The JRFL virus strain was propagated in cultures of peripheral blood mononuclear cells (PBMCs) from adult donors. The IIIB virus strain was propagated in the human T-cell line, Molt-4 and purified from the supernatants, and stored in aliquots at -85C. Virus was concentrated from culture supernatants and purified by pelleting at 110,000 *g* for 90 min. This procedure produces stock virus of between 10^6^ and 10^7^ syncytia forming units per 0.1 ml. The pellets were gently washed and resuspended in medium. The multiplicity of infection (MOI) of the IIIB strain is determined by counting syncytia formed by HIV-infected lymphocytes when co-cultured with exponentially growing CD4-bearing SupT1 cells. The 50% tissue culture infectious dose (TCID_50_) for the M-tropic virus is determined using PBMCs. In all infectivity experiments cells were infected with HIV-1 at a multiplicity of infection (MOI) of 0.1.

### HIV-1 strong-stop expression

The effect of peptide administration on HIV-1 replication in designated experiments was assessed by PCR quantification of strong-stop proviral R/U5 long-terminal repeat structures as described by Cole et al. [14], with minor modification. Briefly, DNA isolated 4 hours after infection was amplified with HIV-1 R/U5 primers 5′-CAAGTAGTGTGTGCCCGTCTGT-3′ and 5′-CTGCTAGAGATTTTCCACACTGAC-3′, which correspond to nucleotides 560-581 of the R region, and nucleotides 612-635 of the U5 region. The PCR amplification of strong-stop DNA was quantitated using DNA isolated from chronically-infected (HIV-1 strain IIIB) human T cells as a standard, using the VB thymocyte cell line. In each case, the DNA isolation efficiency was controlled by quantitation of β-globin DNA. Data were analyzed for statistical significance using one-way analysis of variance.

### HIV-1 5′ LTR expression

The effect of peptide administration on HIV-1 reprlication was also assessed by PCR quantitification of 5′LTR synthesis, using a method described by Szabo et al. [15]. Briefly, DNA was isolated at the designated times following infection, and PCR was used to amplify a 342 bp region of the 5′ LTR of HIV-1 uisng oligonucleotide DNA primers 5′-AGCCTCAATAAAGCTTGCCT-3′ and 5′-CCCCCTGGCCTTAACCGAAT-3′, followed by electrophoresis in 1.5% agarose gel, and transfer to positively charged nylon membranes for Southern blot analysis. An oligonucleotide probe (5′-GGAGAGAGATGGGTGCGGAG-3′) was 5′ end-labeled with [γ-^32^P]ATP (DNA 5′ End-Labeling System, Promega Corporation, Madison, WI). The membrane was incubated for 2 hr at 37°C in a rotary hybridization incubator with prehybridization solution (6X SSC, 5X Denhardt, 0.05% NaPPi, 100 µg/ml herring sperm DNA, and 5–10 µl probe), and washed five times with washing buffer (6X SSC plus 0.05% NaPPi) the next day. The membrane was exposed to Biomax-MR film, and the film was analyzed by autoradiography. Quantitation of the bands was carried out using 10-fold dilutions of the latently-infected ACH-2 cell line.

### p24-based infectivity assay

Cells were treated with designated concentrations of the peptides 15K or 15D, and after 1 h cells were infected with HIV-1_JRFL_ (monocyte tropic) at an MOI of 0.1. After 2 h, cells were washed, and cultured for additional 48–72 h, followed by analysis of HIV replication by determination of accumulated p24 in the supernatant. The production of p24 was determined by a sandwich ELISA, using ELISA plates pre-coated with capture anti-p24 antibodies provided by the AIDS Vaccine Program (SAIC Frederick, NCI-FCRDC, Frederick, MD). The captured p24 antigen was detected using rabbit anti-HIV-1 anti-p24 antibody, and a secondary goat anti-rabbit IgG (peroxidase-labeled) antibody. The captured p24 protein was detected using 3,3′,5,5′-tetramethylbenzidine and hydrogen peroxide detection system (KPL Laboratories, Gaithersburg, MD). The reaction was read spectrophotometrically at 450 nm. Data were analyzed for statistical significance using one-way analysis of variance.

### Flow cytometry analysis of CXCR4 and CCR5 cell surface expression after treatment of cells with peptides

CEMx174 cells were pelleted and resuspended at 10^6^ cells/ml in PBS containing 1% BSA and 0.1% sodium azide (FACS buffer). Cells were then preincubated with designated concentrations of peptides or CXCL12 (1 µg/ml) at 22°C for 60 min. FITC-labeled anti-human CXCR4 antibody (clone 12G5, BD PharMingen) was added to the cells per the manufactures instructions and further incubated at 22°C for 40 minutes. Cells were extensively washed with FACS buffer and analyzed using a FACS Calibur flowcytometer (Becton Dickinson). HEK/CCR5 cells were suspended in Dulbecco's PBS containing 1% FCS and 0.05% NaN_3_ (10^4^ cells in 100 µl) and incubated with or without 5 µg of CCL4 (PeproTech, Rocky Hill, NJ), 0.1 mM peptide 15D or 15K for 45 min at 22°C. Cells were then treated with FITC-conjugated anti-human CCR5 antibody (2D7; BD PharMingen), and incubated for 30 min at 22°C. Cells were washed twice, and analyzed using a FACS Scan flow cytometer (Becton Dickinson).

### Chemokine binding assay

Binding assays were performed with the CCR5-expressing 174xCEM and Sup-T1 cell lines by adding unlabeled competitor (peptide or control chemokine) and radiolabeled chemokine, 0.2 ng/ml ^125^I-MIP-1βor SDF-1 αspecific activity 2000 Ci/mmol, NEN Life Science Products) to cells in a binding medium consisting of RPMI 1640 supplemented with 1% bovine serum albumin, 0.1% sodium azide and 25 mM HEPES pH 8.0. Cells were then incubated at 22°C for 30 min with continuous rotation. After incubation, cells were centrifuged through 10% sucrose in PBS, and the supernatant was aspirated and the cell-associated radioactivity was determined. The data were analyzed by nonlinear regression using GraphPad Prizm 3.0.

### Microscope-based Calcium-flux assay

Microscope based ratiometric analysis was performed as described by Zhang, *et al.* [16] with minor modifications. CXCR4/HEK293 cells were cultured in eight-well chambered cover glass slides (Nunc, IL) for 24–48 hours. Before the experiments, cells were incubated for 45 min with FURA-2 (Molecular Probes) in complete DMEM medium, followed by three washes with calcium saline buffer (138 mM NaCl, 6 mM KCl, 1 mM CaCl_2_, 1 mM MgCl_2_, 5.5 mM glucose, 0.1% bovine serum albumin, pH 7.4). Peptides (15K and 15KS) or CXCL12 were diluted in PBS to appropriate concentrations. Ratiometric calcium imaging was performed using a Nikon Eclipse TE200 fluorescence microscope equipped with a variable filter wheel (Sutter Instruments, Novato, CA), a Spot charge-coupled device camera, and a Nikon S Fluor 40X objective lens. Dual images (340 and 380 nm excitation, 510 emission) were collected by Openlab System 3.14 (Improvision, Lexington, MA) and pseudocolor ratiometric images were monitored every four seconds. Cells were incubated with PBS, 15K (1.8 mM), or 15KS (1.8 mM) for 12 seconds, followed by a stimulation by CXCL12 (15 nM) at 24 seconds.

### Chemokine production in PBMCs

PBMCs were cultured in RPMI-1640 medium supplemented with 10% fetal calf serum, and peptides were added at the designated concentrations. At 24, 48, 72 and 96 h, the supernatants were collected, and tested for the level of CCL2, CCL3, CCL4, CCL5 and CXCL12 by ELISA. Values represent the mean (± SD) of triplicate cultures, and the results are representative of 3 different donors.

## Results

### Amino acid homology between gp120 and chemokines

We noticed that in the amino acid sequences of most CC and some CXC chemokines there is a Trp residue located at the beginning of C-terminal α-helix, and separated by six residues from the fourth Cys residue. In the amino acid sequences of gp120 of all HIV-1 isolates there is a very similar motif in conserved region 3 (C3) adjacent to the V3 loop [17]. Moreover, several residues of the C-terminal part of variable loop 3 (V3) are also conserved between different HIV-1 strains and exhibit some homology with the chemokines (Table 1).

**Table 1:** Amino acid alignment of sequences of gp120 of some HIV-1 strains with CCL4 and CXCL12.

	V3	V3	V3	V3	V3	V3	C3	C3	C3	C3	C3	C3	C3	C3	C3
**HIV-1 strain** a															
HXB2	M	**R**	**Q**	A	H	**C**	N	I	S	R	A	**K**	**W**	N	N
	**326**														**340**
IIIB	M	**R**	**Q**	A	H	**C**	N	I	S	R	A	**K**	**W**	N	A
	**326**														**340**
JRFL	I	**R**	**Q**	A	H	**C**	N	I	S	R	A	**K**	**W**	N	D
	**322**														**336**
Consensus A	I	**R**	**Q**	A	H	**C**	N	V	S	R	S	E	**W**	N	K
	**310**														**324**
Consensus B	I	**R**	**Q**	A	H	**C**	N	I	S	R	A	Q	**W**	N	N
	**322**														**336**
**Chemokine**															
CCL4	S	**K**	**Q**	-	V	**C**	A	D	P	S	E	S	**W**	V	Q
	**47**														**60**
CXCL12	N	**R**	**Q**	-	V	**C**	I	D	P	K	L	**K**	**W**	I	Q
	**46**														**59**

a The residues numbered according to Korber (1998). The residues identical in HIV-1 gp120 and chemokines are shown in bold. The location of the residues relative to the V3 loop/C3 region is noted.

### Computer modeling of HIV-1 gp120 fragment based on the structure of CCR5 ligand CCL4

To assess the possibility that this particular fragment of gp120 may potentially assume a structure similar to that of chemokines, a model of the corresponding structure of gp120 was built using the structure of CCL4 [18] as the template. Under the constraints of the CVFF molecular mechanics force field, such a model of the three-dimensional structure of this gp120 segment could be generated without violation of protein stereochemistry. The resulting structure possessed the same helical character as the protein backbone of CCL4with a root mean square (RMS) deviation of the superimposed backbone atoms of 0.26. In an effort to examine the functional activity of this region we synthesized peptides corresponding to the gp120 sequence for further studies.

### Rationale for design of inhibitory peptides

The synthesized peptides corresponding to sequences of gp120 homologous to chemokines are shown in Table 2. The sequence of HIV-1_JRFL_, which is very close to the consensus B of HIV-1 sequences [17], was used as a template for the design of anti-HIV peptides. Because gp120 of different HIV-1 strains have some sequence variability even in conserved regions, we designed peptides with sequences similar to the widest range of naturally existing variants. In many HIV strains there is a lysine residue instead of glutamine in position 3 (Table 2). Moreover, it was reasonable to change glutamine for lysine in the synthetic peptide to avoid hydrolysis and conversion to glutamic acid near the conserved positively charged arginine. The corresponding peptide was designated 15K. In another peptide the lysine-12 preceding tryptophan was changed to aspartic acid (peptide designated 15D) because some HIV-1 isolates have aspartic acid in this position and we wished to explore the significance of this substitution. For control experiments, a scrambled peptide 15KS with the same amino acid composition as 15K was also employed. In designated control experiments a 15 amino acid peptide GIG, with an amino acid sequence unrelated to gp120 or chemokines, was also used.

**Table 2:** Sequences of synthesized peptides.

Peptide	1	2	3	4	5	6	7	8	9	10	11	12	13	14	15
**15 K**	I	R	K	A	H	C	N	I	S	R	A	K	W	N	D
**15 D**	I	R	K	A	H	C	N	I	S	R	A	D	W	N	D
**15 KS**	K	I	N	S	W	R	A	D	N	I	H	C	K	A	R
**15GIG**	G	I	G	D	P	V	T	C	L	K	S	G	A	I	A

### Effect of the 15K and 15D peptides on HIV-1 infection

**Figure 1:**
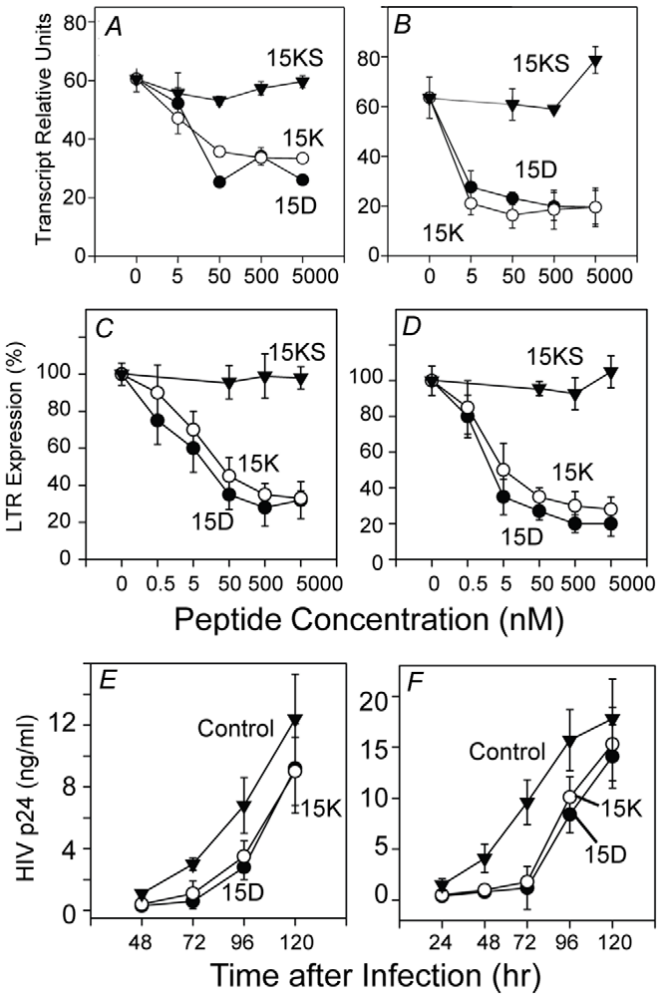
Inhibition of HIV-1 infection by peptides 15K, 15D, and the scrambled peptide 15KS using HIV-1 strain JRFL infection of purified monocytes (Panel A) or PBMCs (Panels C&E), or HIV-1 strain IIIB infection of purified CD4-positive T cells (Panel B) or PBMCs (Panel D&F). Cells were treated with the designated concentrations of peptide for a period of 1 h prior to addition of HIV-1 at an MOI of 0.1, or 500 nM peptide in Panels E&F. Four h after infection the cells were harvested and the HIV early replication was determined by PCR quantification of either strong-stop proviral R/U5 long-terminal repeat structures (Panels A& B), or at 18 hrs for the measurement of the 5′LTR (Panels C&D). The expression of HIV-1 p24 was also determined as a measure of HIV replication (Panels E&F). Results are presented for a representative experiment except for the data in Panels C and D where the collective data for all experiments are presented as the mean ± SD for each data point.

Our computer modeling of the conserved region of gp120 suggested that the peptides 15K and 15D might block the ability of CCR5 to serve as an HIV-1 co-receptor. We determined the capacity of the peptides to inhibit infection of CD14+ monocytes by HIV-1_JRFL_, a monocyte-tropic HIV-1 strain, using PCR quantification of strong-stop proviral R/U5 long-terminal repeat structures [14]. Our results (Figure 1A) show that the addition of either 15K or 15D to the cells reduces infection with HIV-1, with significant inhibition at concentrations as low as 50 nM. The scrambled peptide 15KS with the same amino acid composition as 15K but a randomized sequence (Table 2) manifested no inhibition of virus infectivity demonstrating the importance of the particular amino acid sequence for inhibitory activity. We obtained essentially the same results from experiments in where the expression of the HIV 5′LTR was determined (Figure 1C) following infection of PBMCs.

Based on the homology of the 15K and 15D peptides to the CXCR4 ligand CXCL12, we also wished to determine the capacity of the peptides to inhibit CXCR4 co-receptor function. Experiments were carried out to assess the ability of 15K or 15D to alter the infection of PBMCs with the T cell-tropic HIV_IIIB_ strain. Our results show that both peptides significantly reduced HIV-1 infection in a dose-dependent manner (Figure 1B). Significant inhibition of the HIV-1 infection was detected with concentrations of peptides as low as 5 nM. The scrambled peptide 15KS with the same amino acid composition as 15K but a randomized sequence (Table 2) manifested no inhibition of virus infectivity demonstrating the importance of the particular amino acid sequence for inhibitory activity. Here again, we obtained essentially the same results from experiments in which we determined the expression of HIV 5′LTR following infection of PBMCs. The virus inhibition data suggest that CXCR4-specific virus IIIB is more sensitive to peptides 15K and 15D than CCR5-specific virus JRFL (Figure 1B vs 1A, and Figure 1D vs 1C) probably because of higher affinity of interaction of the peptides with CXCR4 than CCR5. Finally, we also assessed the inhibition of HIV replication in PBMCs over a 5 day period, and the results show that the greatest inhibition is apparent for the first 72 hours following infection (Figure 1E & 1F).

**Figure 2:**
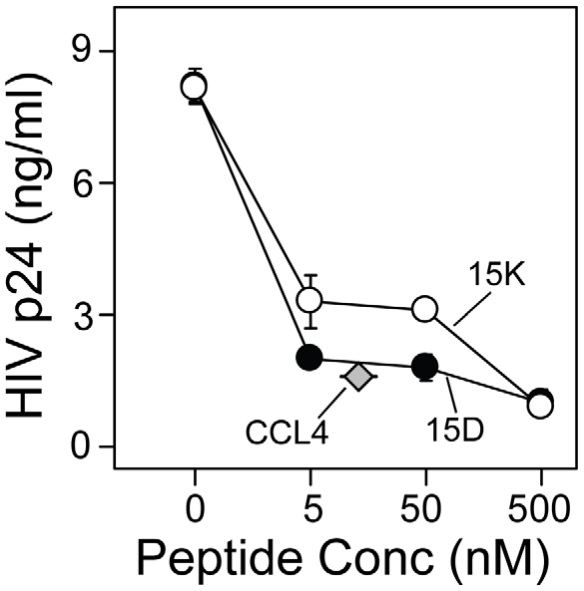
Inhibition of HIV-1 strain JRFL infection of monocyte-derived macrophages by peptides 15K, 15D and chemokine CCL4. Monocyte-derived macrophages were treated with the designated concentrations of peptide for a period of 1 h prior to addition of HIV-1_JRFL_. After 2 hr, cells were washed, and viral replication was determined after 72 hr by p24 analysis. Results are representative of 4 independent experiments. The level of replication in the 15D and 15K groups were significantly different (P<0.01) from the untreated group at concentrations of 5 nM or more.

Since it was reported that CC chemokines effectively inhibit HIV-1 infection of cells [19] we compared the ability of chemokine CCL4 and peptides 15D and 15K to inhibit HIV-1 infection of monocyte-derived macrophages using p24 based infectivity assay. Both peptides exhibited inhibitory activities comparable with CCL4 (Figure 2). Comparison of inhibition of HIV-1 infectivity in monocytes was determined by measuring HIV-1 strong-stop DNA (Figure 1A), and infectivity of macrophages determined by measuring p24 concentrations shows more potent inhibition in the p24 expression assay. This probably reflects the difference between the assays. The detected proviral DNA includes not only full size copies but also shorter fragments which would not lead to synthesis of complete viral RNA and viral proteins; the measurement of p24 level in infected cell supernatants reflects the presence of assembled viral particles.

### Effect of peptides on the binding of antibodies to CXCR4 and CCR5 expressing cells

**Figure 3:**
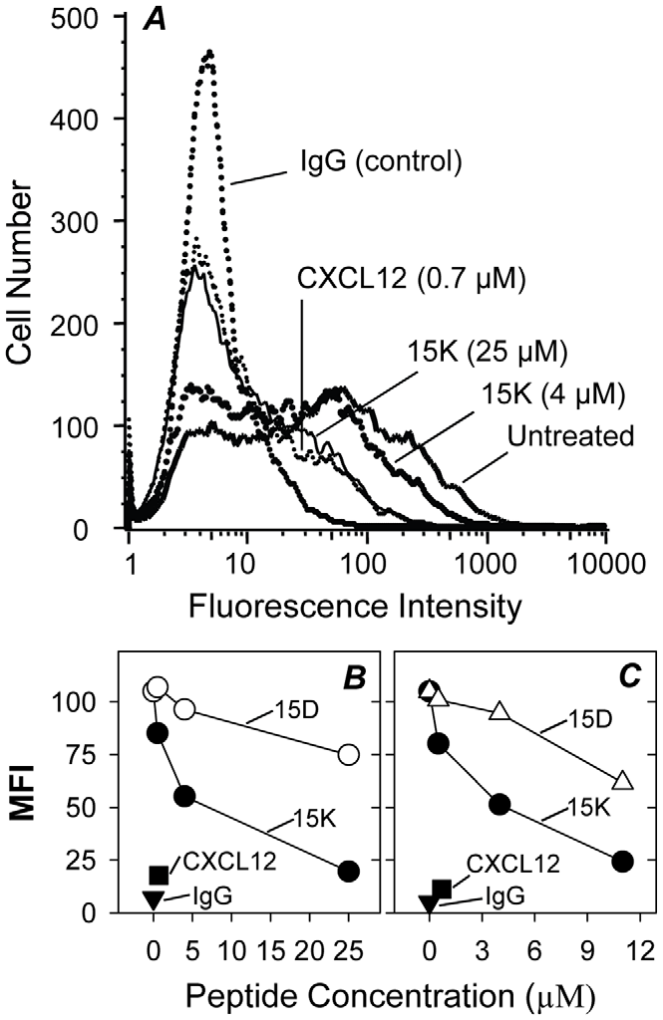
Effect of peptides 15K, 15D and scrambled peptide 15KS on the binding of anti-CXCR4 antibody 12G5 to CEMx174 cells. A. Dose-dependent effect of peptide 15K in comparison with CXCL12. B. The comparison of the effects of 15K and 15D. C. The comparison of the effects of 15K and 15KS. Cells were preincubated at 22°C with peptides at designated concentrations or CXCL12 for 60 min. Then cells were incubated with FITC-labeled anti-human CXCR4 monoclonal antibodies 12G5 for 40 min at 22°C and washed with FACS buffer prior to flow cytometry.

To determine if peptides 15D and 15K interact directly with chemokine receptors we studied the effect of these and control peptides on the binding of anti-chemokine receptor antibodies to CXCR4 and CCR5. The monoclonal antibody 12G5 recognizes a conformational extracellular epitope on CXCR4. This antibody blocks the infectivity of some X4 strains of HIV-1 and HIV-2 [20]-[22]. It also inhibits the binding of CXCL12, a natural CXCR4 ligand [23]. Further, the binding of 12G5 to CXCR4 receptor-expressing cells is prevented by anti-HIV compounds [24]. Our results show that peptide 15K inhibited the binding of the 12G5 antibody to CXCR4 expressing cells in a dose-dependent manner (Figure 3A), and at a concentration of 25 µM approached the efficacy of CXCL12 at 0.7 µM. The more acidic peptide 15D appeared to be less effective in blocking of 12G5 antibody binding to CXCR4 (Figure 3B), which is not surprising since the chemokine binding site of CXCR4 includes several negatively charged residues [25]. To determine the importance of the particular amino acid sequence for the inhibitory activity of the peptides we compared the effects of peptide 15K and the corresponding scrambled peptide 15KS on binding of 12G5 to CXCR4. The scrambled peptide 15KS manifested significantly less inhibitory activity in comparison with 15K suggesting the importance of the specific peptide sequence (Figure 3C).

The monoclonal antibody 2D7 binds to a conformational epitope on CCR5, and inhibits Ca^2+^ flux induced by CCL5. This antibody also inhibits HIV infection in vitro [26] but not the binding of gp120-sCD4 complexes to CCR5 expressing L1.2 murine cells [27]. We tested the effect of peptides 15D and 15K on antibody binding to HEK293/CCR5 by flow cytometry. No significant effect of peptides on anti-CCR5 binding was observed indicating that the peptides bind to an epitope distinct from the antibody-binding site or their binding affinity is not sufficient for effective competition with this particular antibody.

### Effects of peptides on induction of intracellular Ca^2+^ concentration by CXCL12 in CXCR4/HEK293 cells

**Figure 4:**
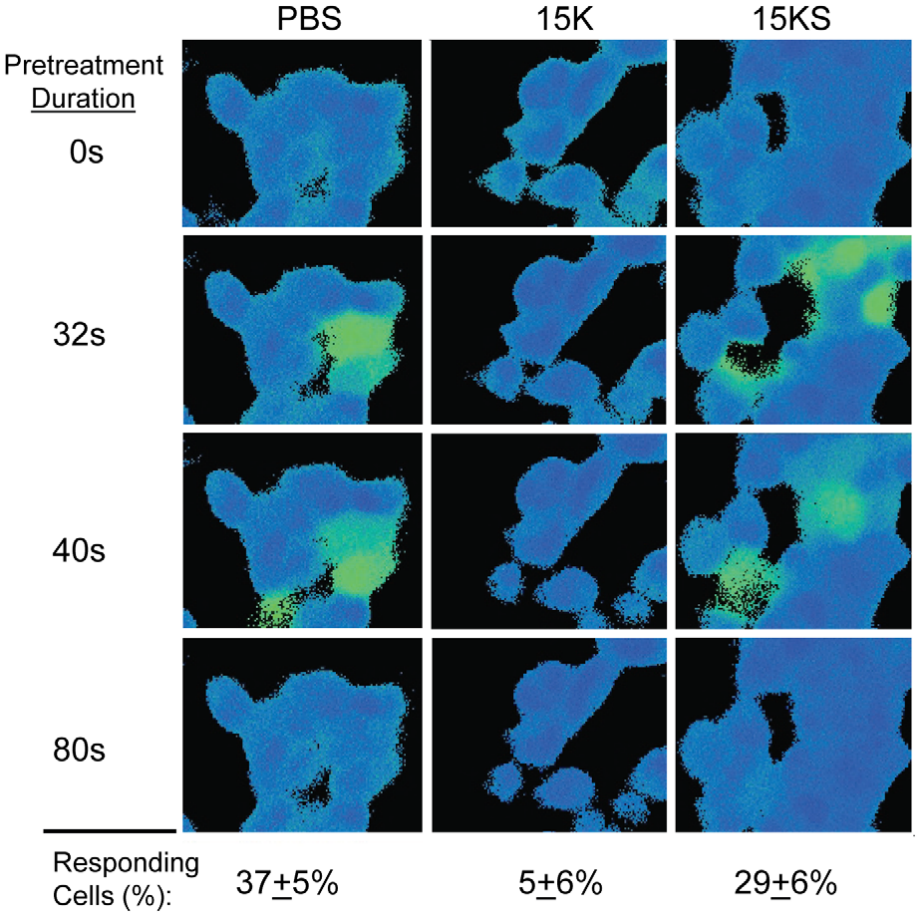
Effect of peptides 15K and 15KS pre-treatment on the mobilization of intracellular Ca^2+^ in CXCR4/HEK cells in response to CXCL12. Cells loaded with Fura-2 were preincubated with peptides at a final concentration of 1.8 mM for 12 sec prior to stimulation with CXCL12 (15 nM).

The increase of intracellular Ca^2+^ concentration mediated by a chemokine receptor in response to a cognate chemokine is a reliable assay for measurement of chemokine agonist activity. CXCR4/HEK293 cells express a high level of CXCR4 and respond well to CXCL12 stimulation, so we used these cells to determine whether peptide 15K could block the activation of CXCR4. Preincubation of cells with peptide 15K (at a concentration of 1.8 mM) for 12 seconds almost completely inhibited the cellular response to CXCL12 implying direct interaction of the peptide 15K with chemokine receptor. The control scrambled peptide 15KS was significantly less effective at inhibiting the Ca^2+^ mobilization response suggesting the importance of the specific peptide sequence (Figure 4).

### Effect of gp120 derived peptides 15 K and 15D on binding of chemokines to CCR5 and CXCR4 expressing cells

**Figure 5:**
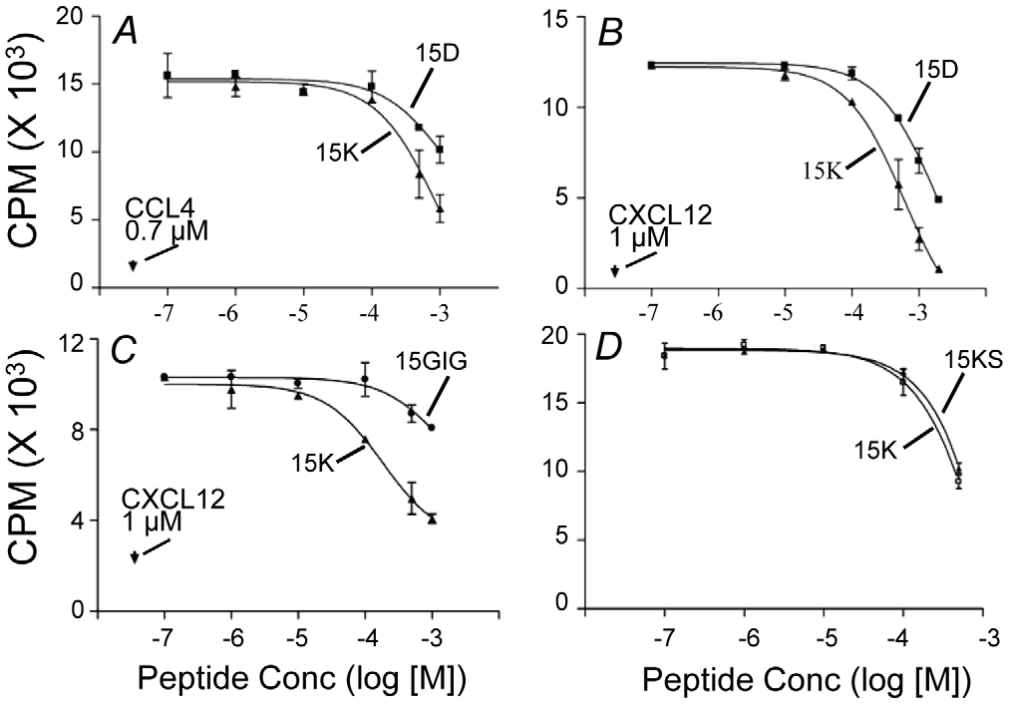
Inhibition of chemokine receptor ligand binding by peptide pre-treatment. (A) Effect of pre-treatment with peptides 15K and 15D on the binding of CCL4 to SupT1/CCR5 cells; (B) Effect of pre-treatment with peptides 15K and 15D on the binding of CXCL12 to CEMx174 cells; and (C) Effect of pre-treatment with 15K and control peptide 15GIG on the binding of CXCL12 to CEMx174 cells; (D) Effect of pre-treatment with peptides 15K and 15KS on the binding of CXCL12 to CEMx174. Binding studies were performed as described in Materials and Methods.

Since the 15K and 15D peptides were designed based on their structural homology with a chemokine fragment, we wanted to determine whether these peptides might exhibit binding activity for CCR5 or CXCR4. Experiments were carried out to assess the ability of the peptides to compete with radiolabeled CCL4 or CXCL12 for binding to cells expressing CCR5 or CXCR4, respectively. To determine the effect of peptides on chemokine binding and to exclude the contribution of chemokine internalization, we incubated cells at room temperature in the presence of 0.1% sodium azide. Under such conditions all cell-bound ligand could be removed by rinsing the cells with 0.1 M Gly-HCl pH 2.5, excluding contribution of internalization to the chemokine binding. The binding experiments show that the peptides 15D and 15K competitively inhibited chemokine binding to CCR5 (Figure 5A) and CXCR4 (Figure 5B) although with low potency. The control 15-mer peptide 15GIG with a unrelated amino acid composition manifested significantly lower competing activity (Figure 5C). The binding experiments did not reveal any significant difference in the inhibitory activity of peptide 15K in comparison with the scrambled analog (Figure 5D).

### Induction of chemokine production in PBMC by peptides 15K and 15D

**Figure 6:**
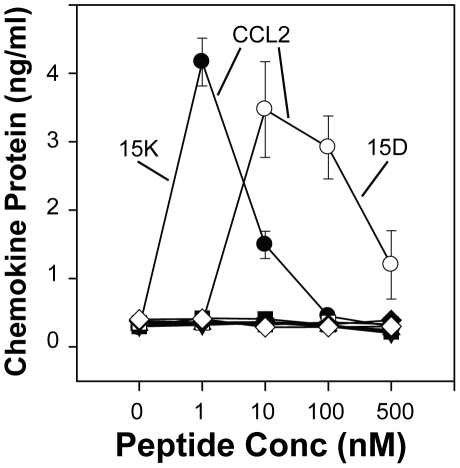
Induction of chemokine expression following peptide administration. Cells were treated with 15K and 15D, at the designated concentrations, and supernatants were collected from the cultures at 48 h, and subjected to ELISA for the determination of CCL2, CCL3, CCL4, CCL5 and CXCL12 levels. Levels of chemokine at 24, 72, and 96 h were also monitored for chemokine levels (data not shown). Data are presented as the mean (± SD) of triplicate determinations, and the data are representative of the results from experiments with three donors.

CCL3, CCL4, CCL5 and CXCL12 are important natural inhibitors of HIV-1 infection, and it possible that the activation of chemokine receptors may stimulate chemokine expression. The production of these chemokines by in the cultures of peptide-treated cells could contribute to the antiviral activity of the peptides. In an effort to address this possibility, cells were treated with peptides 15D and 15K and the chemokine levels were determined by ELISA. Analysis of CCL2, CCL3, CCL4, CCL5 and CXCL12 in the cultures showed that only CCL2 levels were elevated following peptide treatement, and the increase in CCL2 expression was observed primarily at 48 h post-peptide administration (Figure 6). It appears unlikely that the production of chemokines in the peptide-treated cultures could contribute to the antiviral activity of the peptides.

## Discussion

The interaction of HIV-1 envelope glycoprotein gp120 with a chemokine receptor is a prerequisite for viral attachment and entry into the target cell [7]. Monocyte-tropic viruses utilize chemokine receptor CCR5 for cell entry (R5 strains) and T cell tropic virus utilize CXCR4 (X4 strains). The chemokine receptor binding site on gp120 is formed or exposed after binding of CD4 to gp120 [28]. However, the binding of the gp120 of certain HIV-1 strains to the chemokine receptor does not require interaction with CD4 [29], [30]. The V3 loop of gp120 has been identified as the major determinant of cellular tropism and coreceptor specificity [31], [32]. However, the precise region within this 35–37 amino acid loop responsible for the observed coreceptor-usage phenotype has not been established [33]. It was found that synthetic cyclized peptides corresponding to the V3 loop of gp120 of X4 and dual strains of HIV-1 (but not an R5 strain), at micromolar concentration, could prevent binding of anti-CXCR4 antibodies. Some of these peptides at micromolar concentrations inhibited the infectivity of HIV-IIIB [34]. Synthetic polymeric preparations including a putative V3 consensus sequence (GPGRAF) of HIV-1, were found to inhibit HIV-1 infection by an unknown mechanism; however, it is unlikely that this inhibition was due to competition with gp120 binding to the chemokine receptor or CD4 [35]. Although the influence of V3 on HIV-1 coreceptor utilization is well established, other more conservative regions of gp120 are also involved [36].

We reasoned that the localization of the chemokine receptor binding site of gp120 might be identified based on similarity in amino acid sequence with the chemokines. Indeed, we observed that in the amino acid sequences of most chemokines there is a Trp residue separated by six amino acid residues from the fourth Cys residue. The gp120 of all HIV-1 isolates have a similar motif in the C3 region following the V3 loop. Moreover, several residues of the C-terminal part of the V3 loop also manifest some homology with the chemokine sequence. Our computer modeling demonstrated that this fragment of gp120 could be template forced onto the homologous loop of the known three-dimensional structure of CCL4 without violation of protein stereochemistry.

**Figure 7:**
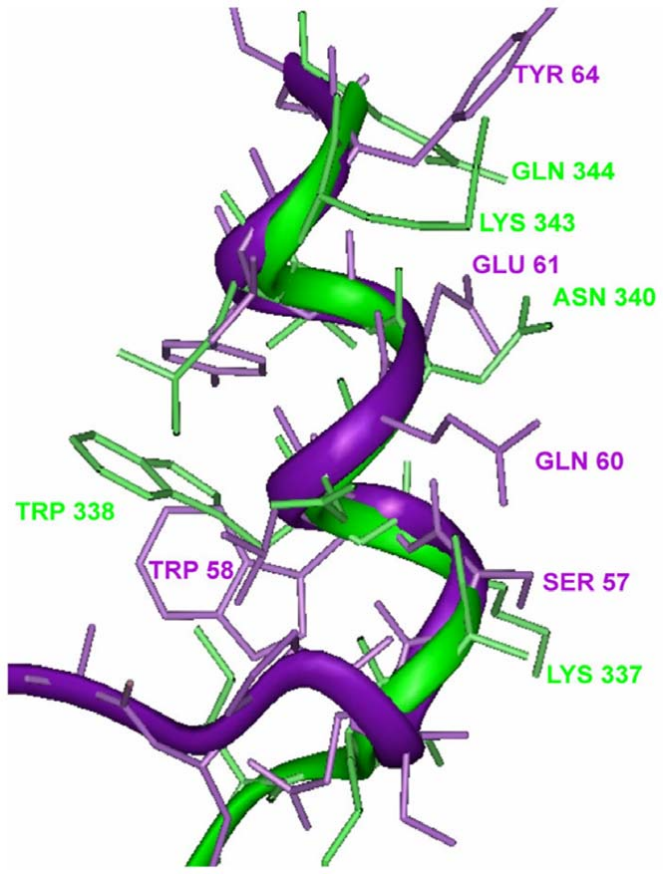
Comparison of the structures of the fragments of gp120 (HIV_HXB2_ residues 331–344, PDB code 1gc1) and CCL4 (residues 47–64, PDB code 1hum). Gp120 is shown in green and CCL4 is in purple. The solvent interface in the context of both of the whole proteins is to the right of the figure. The gp120 fragment was rotated and side chain residues were allowed to move within the constraints of the CVFF mechanics employed (see Methods: Computer modeling) in order to align homologous amino acids. Both structures exhibit a buried Trp residue at a turn preceding an α-helix which has polar side chains on the surface. The location of the Trp in the start of the helix and the hydrophobic nature of the residues surrounding the Trp are also conserved features in both structures.

Importantly, our hypothesis that this particular fragment of gp120 may interact with chemokine receptor similarly to a chemokine is in agreement with published crystal structures of gp120 and chemokines. The X-ray structure of an HIV-1 gp120 core (not including V-loops), complexed with a two-domain fragment of CD4, and an antigen-binding fragment of neutralizing antibody, was reported [5]. Although the structure does not include variable loops of gp120 it did include the C3 portion of the fragment of gp120, which we considered to be structurally similar to the chemokines and potentially involved in interaction with chemokine receptors. In fact, alignment of the three-dimensional structure of this fragment (HIV_HXB2_, residues 331–340, Table 1) with the corresponding fragment of CCL4 demonstrates that they are similar (Figure 7). Both motifs are characterized by an α-helix preceded by a turn. Both contain a Trp residue with the indole ring buried in the interior region of the turn and with the α-carbon present on an exterior turn of the helix. The surface residues on the helices are characterized by long aliphatic side chains, terminated with polar groups, including Lys, Glu, Gln, and Asn. Moreover, this fragment is located within the region of gp120 molecule which was implicated in CCR5 binding [36].

Interestingly, when Sharon, et al. [37], determined the structure of V3-MN peptide bound to Fv fragment of broadly neutralizing monoclonal antibody 447-52D by NMR, it was found to have conformational similarity to β hairpins in CCL4 and CCL5; the structure of V3-IIIB peptide was similar to CXCL12. They hypothesized that these structural differences in V3 loop of gp120 dictate which chemokine receptor a virus binds. The sequence corresponding to our peptides is more conservative and might be interacting with the sites of chemokine receptors, which are similar between CC and CXC chemokine receptors.

At present it is assumed that the N-terminal region and the so called N-loop following the first two cysteine residues of the chemokines play the most important role in interaction with the chemokine receptors, although apparently other parts of these molecules contribute to receptor binding [38]–[40]. The C-terminally truncated form of IL-8 missing the α-helix and β-turn manifested greatly reduced chemotactic activity [38]. Additional confirmation of the significance of this segment of chemokines for receptor activation is provided by the observation that substitution of Arg-45 and Arg-47 with Ser reduced the biological activity of human CCL4 [41]. Finally, a peptide corresponding to the CCL-2 sequence just preceding the C-terminal α-helix, and its derivatives, inhibited the recruitment of leukocytes to inflammatory sites in animal models manifesting properties of broad spectrum chemokine inhibitors [42]. This may imply that this site in chemokines is responsible for some important yet unknown step in chemokine function *in vivo.* In a recent study it was demonstrated that the residues including the beginning of the C-terminal α-helix of CXCL12 could be involved in interaction with CXCR4 [43].

Since the peptides 15D and 15K include only 5 residues from the C-terminal part of the V3 loop, which is more conserved than other parts of V3 loop, and 9 residues from the N-terminal part of the conservative C3 region, it was reasonable to expect that anti-HIV activity of these peptides may not be dependent on virus tropism. The effects of the peptides on infectivity of R5 and X4 viruses confirmed this prediction.

Our finding that peptides 15K and 15D compete with anti-CXCR4 antibody 12G5 suggests a direct interaction of the peptide with this receptor. Although the 15D peptide had reduced ability to block binding of the 12G5 antibody, probably because it has a negative Asp residue instead of Arg and it is known that the chemokine binding site of CXCR4 is negatively charged, the infectivity data indicate that the low-affinity interaction of 15D was still sufficient to interfere with viral infection. It was established [44] that MIP-1β and HIV-1 gp120 have overlapping but distinct binding sites. Thus it is possible that our peptides based on gp120 sequence can more effectively compete with HIV-1 gp120 attached to virus than with the chemokine ligands. Moreover, binding to CD4 induces a conformational change in virus-associated gp120, which allows it to interact with chemokine receptor [28]. This conformational state is very likely to be transient, avoiding generation of neutralizing antibodies, and has a limited time for productive interaction with the chemokine receptors. Consequently, if the binding site for HIV-1 gp120 on a chemokine receptor is occupied by our peptides it may have strong inhibitory effect on virus infectivity. Apparently our peptides are not strong competitors of chemokine binding, as indicated by radio-labeled binding analysis, but have a significantly slow rate of dissociation from receptor which would be consistent with our calcium mobilization results, and would also be consistent with the weak ability of chemokine to displace inhibitory peptide from receptor immediately when chemokine is added. We think that these two factors may explain rather strong antiviral and low chemokine antagonistic activity of peptides. Of course more studies are necessary to fully understand the mechanism of the antiviral activity of these peptides.

When ligands such as free chemokines or HIV-1 gp120 interact with chemokine receptors they can induce expression of additional chemokines by the cells [45]–[47]. Thus a possible explanation for potent inhibition of virus infectivity by our peptides could be through the induction of chemokine production, which could potentially inhibit virus attachment and infectivity. We failed to detect the induction of CCL3, CCL4, CCL5 or CXCL12 following peptide administration (Figure 6). For this reason, we find it unlikely that chemokine induction from the target cells by the peptides during the infectivity assays played a significant role in the observed antiviral activity.

The affinity of interaction of the peptides 15D and 15K with chemokine receptors is apparently low. However, even this low affinity interaction is sufficient for interference with HIV-1 infection based on the low concentration of peptides required for blocking of the viral infection of macrophages and T lymphocytes. The use of low affinity anti-viral drugs interfering with HIV-1 coreceptor interaction may allow targeting multiple cellular coreceptors while maintaining the ability to inhibit interactions of a viral glycoprotein, which is subject to frequent mutation. Moreover, the rather weak competition of peptides 15D and 15K with chemokines for receptor binding, together with potent inhibition of HIV-1 infectivity, may be therapeutically preferable to high affinity inhibitors of CXCR4 and CCR5, because these peptides would not compromise functions of potentially critical chemokines such as CXCL12 or the CCR5 ligands.

In conclusion, we believe that we identified a region of HIV-1 gp120, which appears to be structurally similar to chemokines, and is probably directly involved in the interaction with certain chemokine receptors. Our findings that the corresponding peptides inhibit HIV-1 infection of human monocyte-derived macrophages and T-lymphocytes at low nanomolar concentration suggest that these peptides and their analogs may be used to dissect gp120 interactions with different chemokine receptors and could serve as leads for design of new peptide based inhibitors of HIV-1 not restricted by viral tropism [48]. Moreover, it may also be possible that the antibodies raised against this sequence of HIV-1 gp120 may have anti-HIV protective and therapeutic activity.
